# Low expression of NLRP1 is associated with a poor prognosis and immune infiltration in lung adenocarcinoma patients

**DOI:** 10.18632/aging.202620

**Published:** 2021-03-03

**Authors:** Edward Shen, Ying Han, Changjing Cai, Ping Liu, Yihong Chen, Le Gao, Qiaoqiao Huang, Hong Shen, Shan Zeng, Min He

**Affiliations:** 1 Department of Oncology, Xiangya Hospital, Central South University, Changsha 410008, Hunan, China; 2 Department of Life Sciences, McMaster University, Hamilton, ON L8S 4L8, Canada; 3 Key Laboratory for Molecular Radiation Oncology of Hunan Province, Xiangya Hospital, Central South University, Changsha 410008, Hunan, China; 4 National Clinical Research Center for Geriatric Disorders, Xiangya Hospital, Central South University, Changsha 410008, Hunan, China

**Keywords:** LUAD, NLRP1, biomarkers, bioinformatics, immune infiltrate

## Abstract

NLRP1 (NLR family, pyrin domain containing 1), the first NLR protein, described to form an inflammasome, plays critical roles in innate immunity and inflammation. However, NLRP1 has not been reported to be linked to LUAD (lung adenocarcinoma) risk, prognosis, immunotherapy or any other treatments. This research aimed to explore the prognostic value and mechanism of NLRP1 in LUAD. We performed bioinformatics analysis on LUAD data downloaded from TCGA (The Cancer Genome Atlas) and GEO (Gene Expression Omnibus), and jointly analyzed with online databases such as TCGAportal, LinkedOmics, TIMER, ESTIMATE and TISIDB. NLRP1 expression of LUAD tissue was considerably lower than that in normal lung tissue. Decreased NLRP1 expression of LUAD was associated with relatively high pathological, T and N stages. Kaplan-Meier survival analysis indicated that patients with low NLRP1 expression had a worse prognosis than those with high expression. Multivariate Cox analysis further showed that NLRP1 expression level was an independent prognostic factor of LUAD. Moreover, the level of NLRP1 expression was positively linked to the degree of infiltration of various TIICs (tumor-infiltrating immune cells). Our findings confirmed that reduced expression of NLRP1 was significantly related to poor prognosis and low degree of immune cell infiltration in LUAD patients.

## INTRODUCTION

Lung cancer is the leading cause of cancer-related mortality globally, making it one of the urgent health problems [1]. During the past decades, LUAD (lung adenocarcinoma) has become the most prevalent subtype [2]. Despite improvements in cancer-related treatment technology, the five-year survival rate for lung cancer is extremely poor, mainly because the high percentage of patients are diagnosed at an advanced stage, including locally advanced or extensive metastatic stage [3, 4]. Generally, the five-year overall survival rate for patients diagnosed with advanced LUAD is just 15%; however, more than 60% of LUAD patients missed the period in which gene alterations can be directly targeted, which could improve their survival rate [5, 6]. Therefore, discovering accurate and sensitive biomarkers is imperative for improving LUAD outcomes. The rise and great efficacy of immunotherapy have made immune-related biomarkers even more valuable.

Inflammasome signaling has become an emerging pillar of innate immunity and plays crucial roles in regulating health and disease. Over the past 10 years, the roles of inflammasome in host defense against invading pathogens; autoinflammatory, metabolic, and neurodegenerative diseases; and even cancer development have been accepted [7]. Inflammasome complexes are assembled under the activation of certain nucleotide-binding domain, leucine-rich repetitive sequence-containing proteins (NLRs), AIM2-like receptors (ALRs) or pyran [8]. NLR family members participate in innate immune signaling pathways via the activation or inhibition of the inflammasome. Dysregulation of NLR family members results in various inflammatory diseases and autoimmune disorders [9]. Of the NLRs, NLRP1, NLRP3, NLRC4 and NLRP6 impact on the pathogenesis of cancer by regulating innate and adaptive immune responses, cell death and proliferation [10]. In human populations, NLRP1 has been linked to various diseases connected with dysfunctional immunoregulation, including systemic lupus erythematosus, type I diabetes, and vitiligo [11–13], and has also been reported to play key roles in modulating tumorigenesis and evolution [14, 15].

Here, we analyzed NLRP1 data of LUAD patients in TCGA (The Cancer Genome Atlas) and GEO (Gene Expression Omnibus). Using multi-omic analysis, we assessed the clinical parameter alterations and functional networks associated with NLRP1 in LUAD and explored the features of NLRP1 in antitumor immunity. Our results revealed the potential of NLRP1 as a novel target for diagnosis and immunotherapeutic treatment of LUAD.

## RESULTS

### Decreased expression of NLRP1 in LUAD

**Figure 1:**
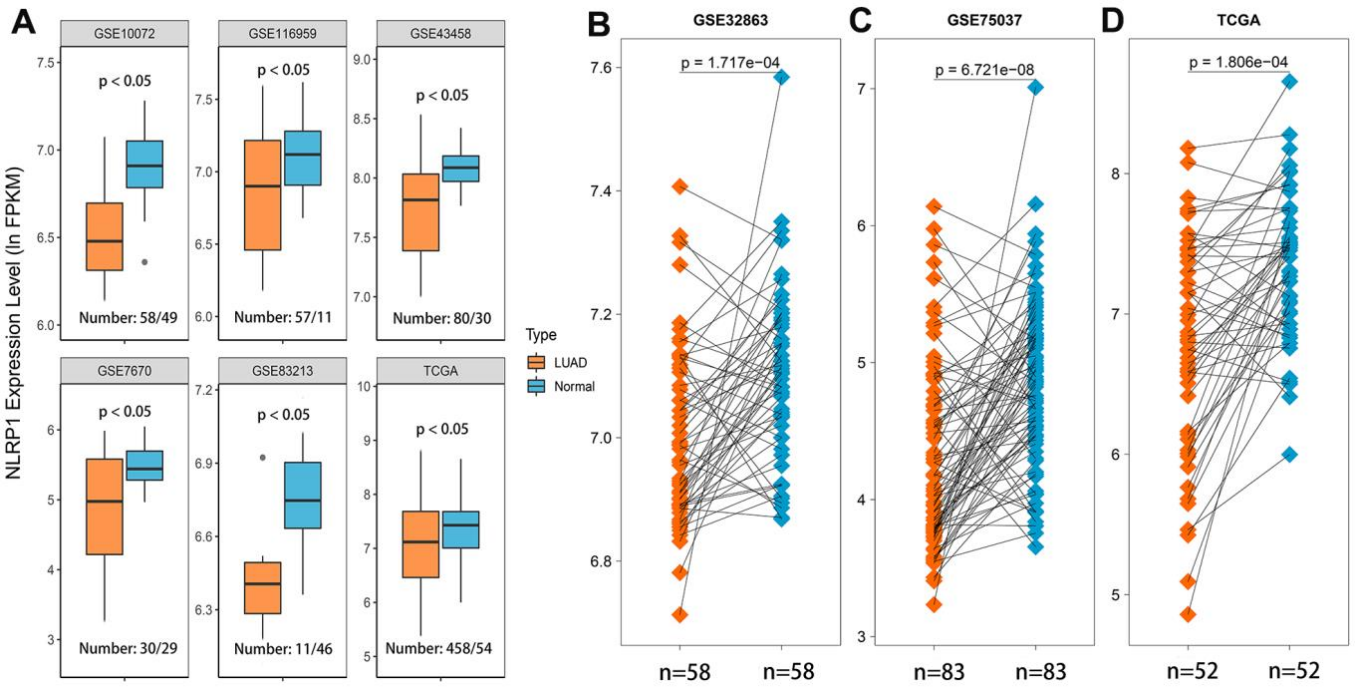
**NLRP1 expression was significantly lower in LUAD tissues than in normal or adjacent normal tissues.** (**A**) NLRP1 expression was significantly down-regulated in cancer tissues compared to normal tissues (*p < 0.05*). (**B–D**) NLRP1 was expressed at a lower level in LUAD tissues than noncancerous adjacent tissues (*p < 0.05*) with the comparison of 192 paired samples from three different LUAD datasets.

We first assessed NLRP1 expression level of six LUAD datasets from the GEO and TCGA databases using the Wilcoxon rank sum test, and the results showed that NLRP1 was expressed at a lower level in LUAD tissues than in noncancerous tissues (Figure 1A, *p < 0.05*). In order to explore whether the above conclusion can be applied to one same patient, we further investigated NLRP1 expression level of 192 LUAD patients tissues and matched para-cancerous tissues in the GEO and TCGA databases. Using the Wilcoxon signed-rank test, we found that NLRP1 was indeed obviously expressed at a lower level in the LUAD tissues (Figure 1B–1D, p <0.05).

### Relationship between NLRP1 expression and clinicopathological parameters in LUAD patients

**Figure 2:**
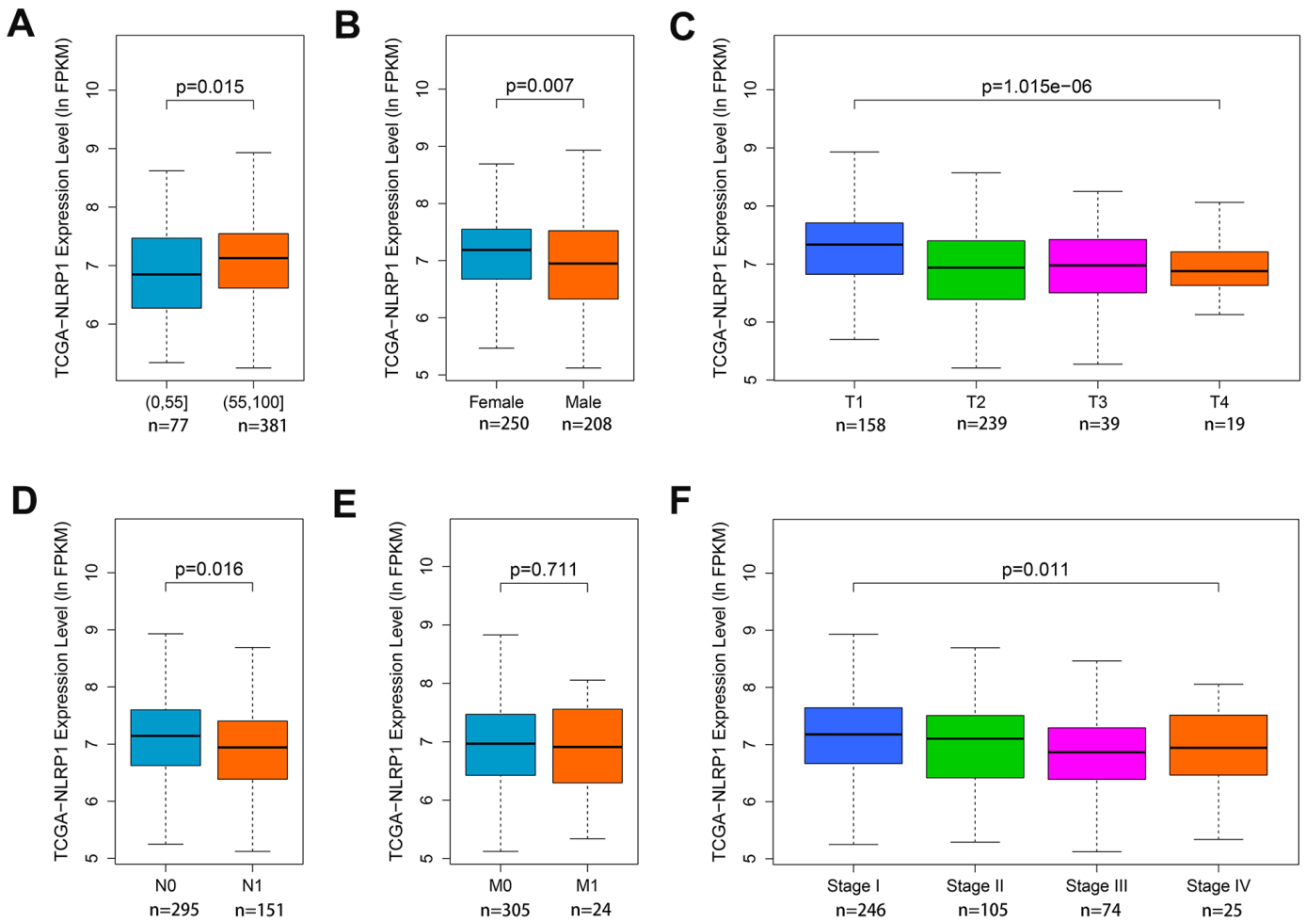
**Associations of NLRP1 expression with clinical parameters.** (**A**) Age (*p < 0.05*); (**B**) Gender (*p < 0.05*); (**C**) T stage (*p < 0.05*); (**D**) N stage (*p < 0.05*); (**E**) M stage (*p > 0.05*) and (**F**) TNM stage (*p < 0.05*).

Since the function of NLRP1 in lung cancer is still unclear, it is necessary to analyze the relationships between NLRP1 expression and some clinical parameters in LUAD patients. To explore the role of NLRP1 in LUAD progression, the Wilcoxon rank sum test or Kruskal-Wallis rank sum test method was applied to analyze the difference of NLRP1 expression level associated with clinicopathological features. Analysis of LUAD data from TCGA database indicated that with the increase in age, the expression level of NLRP1 also elevated (Figure 2A, *p < 0.05*), and the NLRP1 expression level in female sample was higher than that in male sample (Figure 2B, *p < 0.05*). Moreover, results also revealed the significant association between NLRP1 down-regulation and T stage, N stage, and TNM stage (Figure 2C, 2D, 2F, *p < 0.05*).

To further explore whether the expression level of NLRP1 has the same guiding significance as the abovementioned clinical parameters for clinicopathological work, we used Cox regression to analyze the prognostic role of NLRP1 expression level in LUAD. Univariate analysis showed that low NLRP1 expression was related to relatively poor OS (overall survival) (Table 1, *p < 0.05*). Clinical parameters, such as the advanced T stage, N stage, M stage and TNM stage, were all linked to relatively poor OS (Table 1, *p < 0.05*). To further verify the prognostic value of NLRP1 in LUAD, multivariate analysis was performed. The result revealed that only NLRP1 expression and the N stage were independently associated with OS (Table 1, *p < 0.05*). All p-values used the FDR (false discovery rate) to correct for statistical significance of multivariate analysis (Supplementary Table 1). FDR significance level was set at 0.05.

**Table 1:** Univariate and multivariate cox regression analyses for OS in LUAD patients.

**Variables**	**Univariate analysis**			**Multivariate analysis**		
	**No. of patients**	**HR (95% CI)**	***p*-value**	**No. of patients**	**HR (95% CI)**	**p-value**
Age						
≤55 vs. >55	77/367	0.969(0.787−1.193)	0.765			
Gender						
Female vs. Male	244/200	1.086(0.790−1.492)	0.613			
T stage						
T1/2 vs.3/4	387/55	1.533(1.241−1.894)	<0.001	265/41	1.255(0.967−1.629)	0.087
N stage						
N0 vs. N1	286/147	2.818(2.037−3.898)	<0.001	195/111	2.123(1.348−3.345)	0.001
M stage						
M0 vs. M1	295/23	1.994(1.137−3.497)	0.016	286/20	1.121(0.570−2.206)	0.741
Pathological stage						
Stage I/II vs. III/IV	339/97	2.707(11.936−3.786)	<0.001	231/75	1.521(0.880−2.629)	0.133
NLRP1						
High vs. low expression	222/222	0.694(0.555−0.868)	0.001	153/153	0.713(0.542−0.939)	0.016

OS, Overall Survival.

### Relationship between NLRP1 expression and other parameters

**Figure 3:**
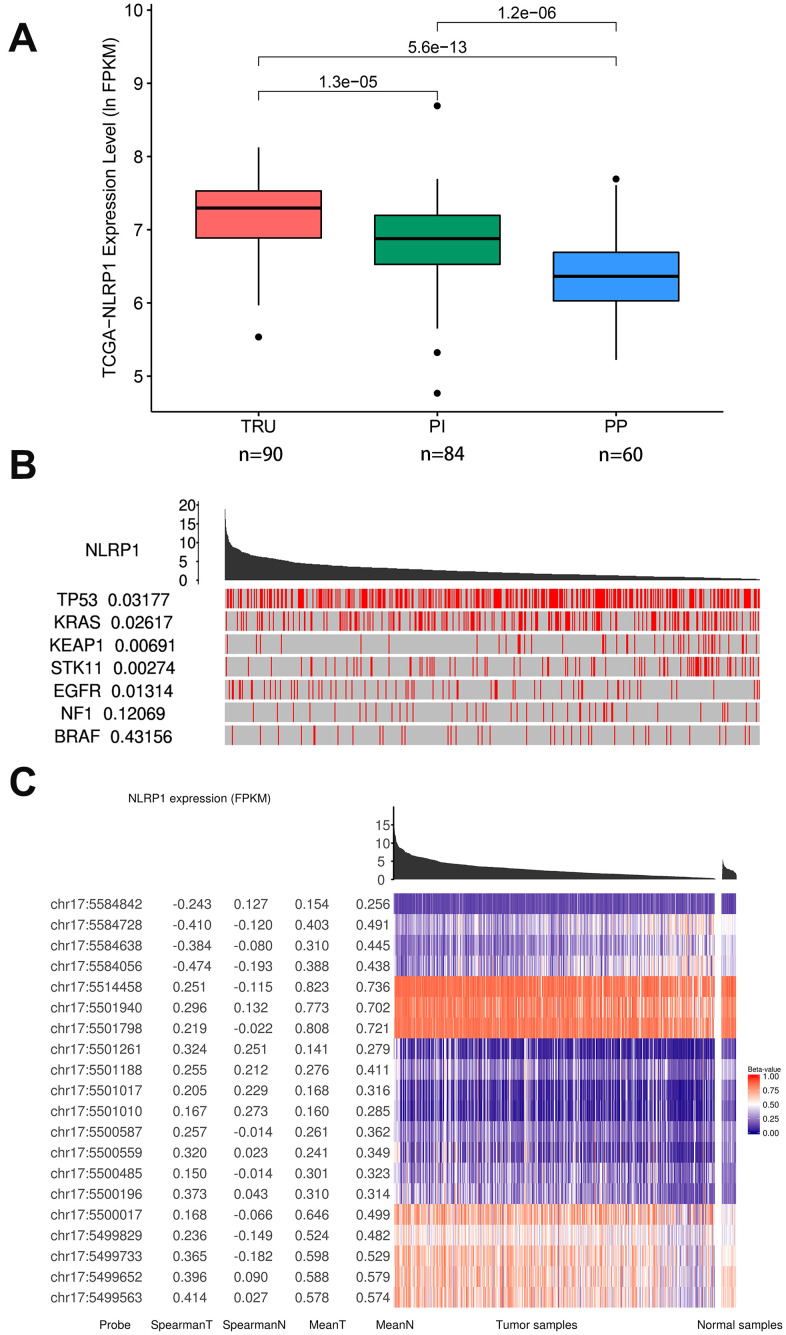
**Relations of NLRP1 expression with other parameters (TCGAportal).** (**A**) NLRP1 expression in the new different subgroups of LUAD. The subgroup of PP had a significantly lower expression level. (**B**) The mutation status of several cancer-related genes with high mutation probabilities in LUAD correlated highly with the expression of NLRP1. (**C**) The methylation levels at multiple sites on chromosome 17 correlated highly with NLRP1 expression. TRU, terminal respiratory unit; PI, proximal inflammatory; PP, proximal proliferative; chr 17, chromosome 17.

In the new subgroup system reclassified by histopathology, anatomy and mutation classification of LUAD, the subgroups of TRU (terminal respiratory unit) and PI (proximal inflammatory) had higher expression levels of NLRP1 than the PP (proximal proliferative) subgroup (Figure 3A).

In addition, samples with lower expression level of NLRP1 were enriched with mutations in TP53, KRAS, KEAP1 and STK11. The elevation of NLRP1 expression level more appeared in samples with EGFR mutation (Figure 3B). So the NLRP1 expression level was significantly affected by the mutation status of several cancer-related genes with high mutation probability in LUAD. These influences were well confirmed in the GSE72094 dataset (missing KEAP1 mutation data) (Supplementary Figure 2).

Moreover, Figure 3C clearly shows that samples with low expression level of NLRP1 were closely related to the higher DNA methylation level on multiple sites of chr17: 5584728, 5584638, 5584056, etc. Besides, the lower DNA methylation level on multiple sites of chr17: 5499563, 5499652 and 5499733 can also influence the expression level of NLRP1.

### NLRP1 coexpression networks in LUAD

**Figure 4:**
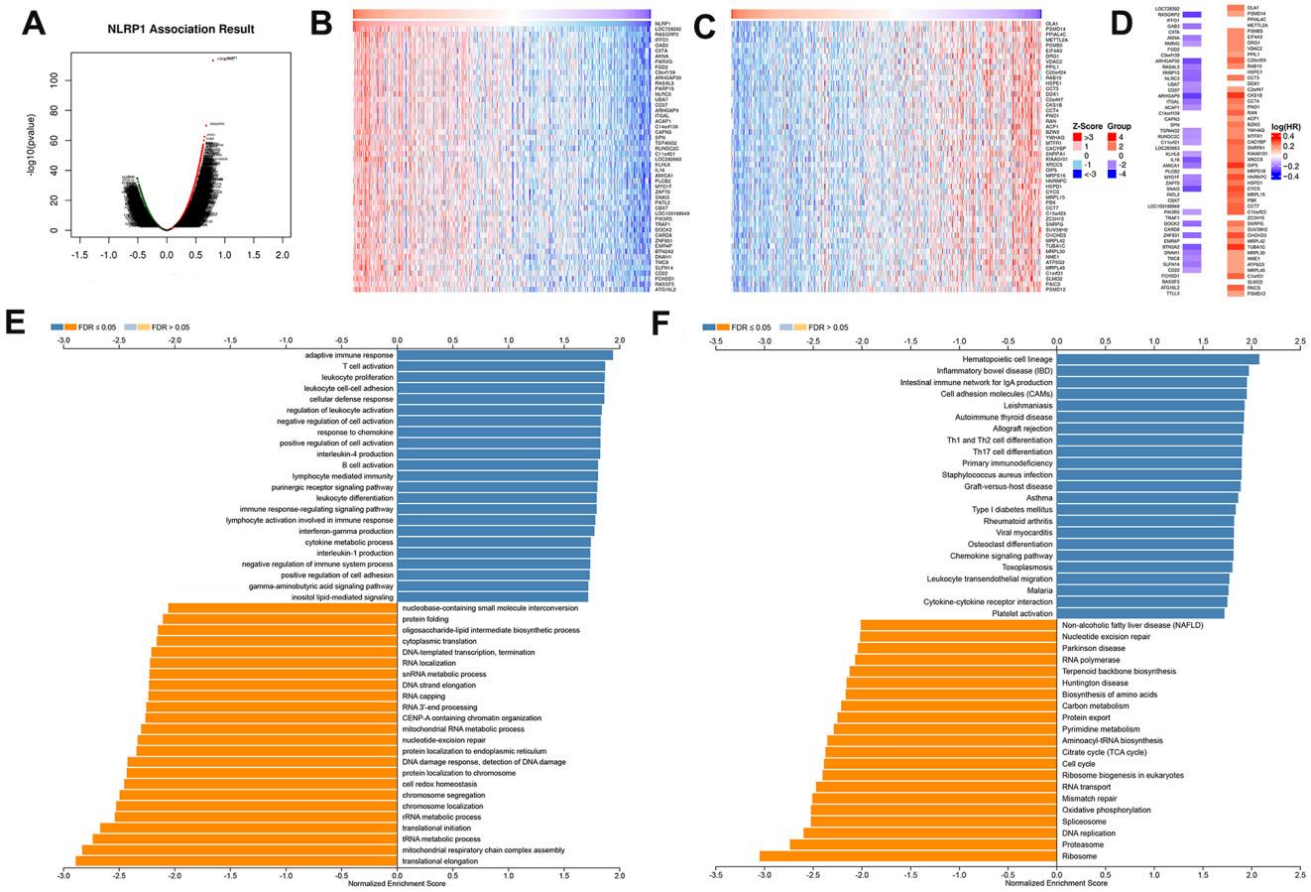
**NLRP1-coexpressed genes in TCGA-LUAD cohort (LinkedOmics).** (**A**) All genes highly associated with NLRP1 identified by Pearson correlation test in the LUAD cohort. (**B**, **C**) Heatmaps respectively show the top 50 genes positively and negatively correlated with NLRP1 in LUAD. Red indicates positively correlated genes, and blue indicates negatively correlated genes. (**D**) Survival map of the top 50 genes positively or negatively correlated with NLRP1 in LUAD. (**E**, **F**) Significantly enriched GO annotations and KEGG pathways of NLRP1 in LUAD cohort. FDR: false discovery rate.

To investigate the mechanism of NLRP1 regulating LUAD progression, the NLRP1 co-expression network of the TCGA-LUAD cohort was constructed with the functional module of the LinkedOmics database. The volcano plot of Figure 4A shows that 8,479 genes (dark red dots) were significantly positively correlated with NLRP1, and 4,401 genes (dark green dots) were significantly negatively correlated. Figure 4B, 4C respectively show the top 50 significant genes positively and negatively associated with NLRP1. Notably, the top 50 significantly positively related genes showed a high likelihood of being low-risk genes, such as NLRP1, and 30/50 genes had a low HR (hazard ratio) (*p < 0.05*). In contrast, there were 44/50 genes with a high HR (*p < 0.05*) in the top 50 significantly negatively related genes (Figure 4D).

Significant GO term annotation by GSEA showed that NLRP1-coexpressed genes were mainly enriched in the adaptive immune response, T cell activation, leukocyte cell-cell adhesion, and regulation of leukocyte activation, while activities such as the tRNA metabolic process, translational initiation, and translational elongation were inhibited (Figure 4E).

KEGG pathway analysis showed enrichment in the hematopoietic cell lineage, inflammatory bowel disease (IBD), leishmaniasis, intestinal immune network for IgA production, cell adhesion molecules (CAMs), Th1 and Th2 cell differentiation, Th17 cell differentiation, staphylococcus aureus infection and nucleotide excision repair pathways (Figure 4F).

### Correlations between NLRP1 expression and immune infiltration levels

**Figure 5:**
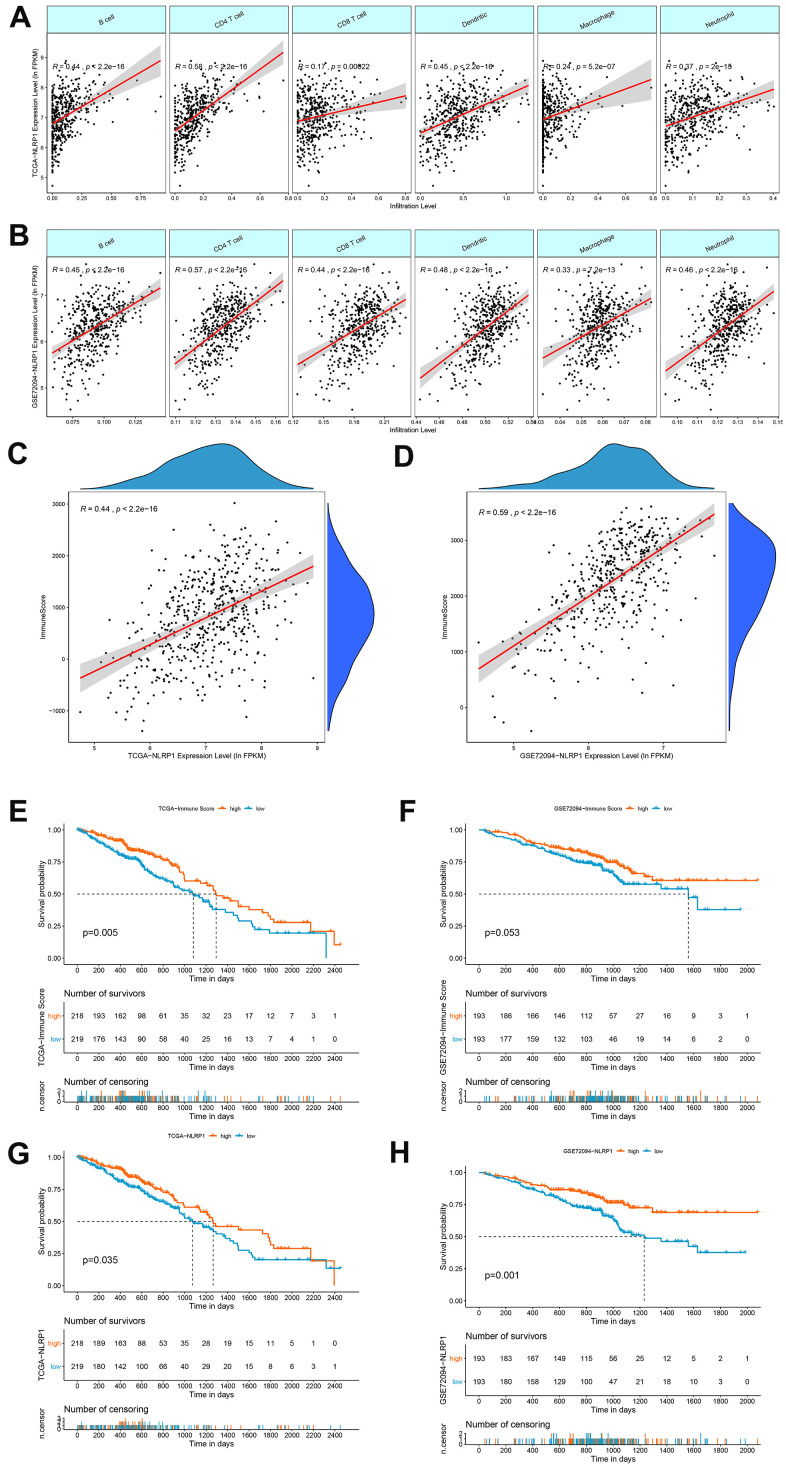
**Correlations of NLRP1 expression with immune infiltration levels in LUAD.** (**A**, **B**) The expression levels of NLRP1 in the TCGA-LUAD and GSE72094 datasets had significant positive correlations with the infiltration levels of B cells, CD4 T cells, CD8 T cells, dendritic cells, macrophages and neutrophils. (**C**, **D**) The expression of NLRP1 had a significant positive correlation with the immune score of LUAD samples based on the ESTIMATE algorithm in the TCGA-LUAD and GSE72094 datasets. (**E**, **F**) Kaplan-Meier survival curve analysis showed that patients with a higher immune score had longer overall survival time than those with a lower immune score in the TCGA-LUAD and GSE72094 datasets. (**G**, **H**) Kaplan-Meier survival curve analysis showed that patients with a higher NLRP1 expression level had longer overall survival time than those with a low expression level in the TCGA-LUAD and GSE72094 datasets.

We explored whether the level of NLRP1 expression is linked to various immune cell infiltration levels in LUAD in data from the TIMER database. Spearman rank correlation analysis showed significant positive correlations between NLRP1 expression and B cells, CD4 T cells, CD8 T cells, dendritic cells, macrophages and neutrophils (Figure 5A). The positive correlation between NLRP1 expression and these immune cells observed in the TCGA-LUAD dataset was well confirmed in the GSE72094 dataset (Figure 5B).

We then used the ESTIMATH algorithm to analyze whether NLRP1 expression was related to the total immune infiltration level in LUAD. The results showed a positive correlation between NLRP1 expression and the immune score in both the TCGA dataset and the GEO LUAD dataset (Figure 5C, 5D). Moreover, patients with higher immune score had a better prognosis than patients with lower immune score (Figure 5E, 5F). And patients with higher NLRP1 expression level had a better prognosis than patients with lower NLRP1 expression level (Figure 5G, 5H).

### Correlations between NLRP1 expression and immune molecules

To broaden the understanding of the correlations between NLRP1 and immune infiltration, we investigated the connection between NLRP1 expression and various immune signatures, including the immune-related signatures of 28 tumor-infiltrating lymphocytes from Charoentong’s study, three kinds of immunomodulators, chemokines and receptors.

**Figure 6:**
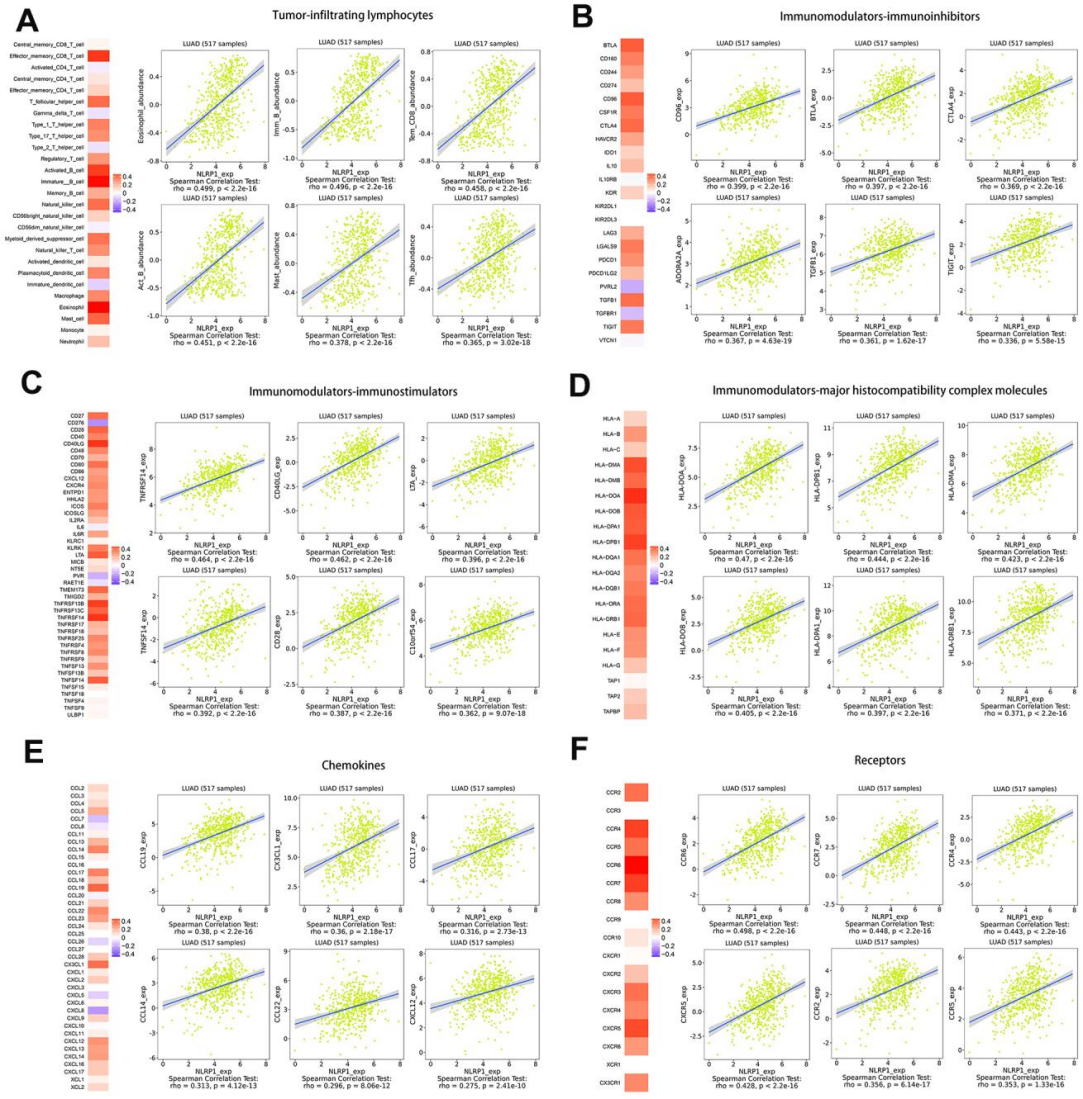
**Spearman rank correlation analysis of NLRP1 with lymphocytes, immunomodulators and chemokines (TISIDB).** (**A**) Relations between the abundance of tumor-infiltrating lymphocytes (TILs) and NLRP1 expression (plus the six TILs with the highest correlations). (**B**–**D**) Relations between three kinds of immunomodulators and NLRP1 expression (plus the six immunomodulators with the highest correlations). (**E**, **F**) Relations between chemokines (or receptors) and NLRP1 expression (plus the six chemokines (or receptors) with the highest correlations).

Spearman rank correlation between NLRP1 expression and various immune signatures was firstly obtained from the TISIDB database. We then evaluated the correlations between NLRP1 and the molecules of all six types of immune signatures in the form of heatmap, and specifically listed the six highest correlated molecules of every type (Figure 6).

## DISCUSSION

NLRP1, the first NLR protein described to form an inflammasome, whose role in malignancy was highly complex. The NLRP1 inflammasome could serve an instigator of chronic myeloid leukemia, melanoma, osteosarcoma, breast cancer, and prostate cancer [16–20]. Hyper-activation NLRP1 inflammasome led to decreasing of tumor cells’ death and increased inflammatory microenvironment driven by IL-18 and IL-1β secretion, which has been proposed as a common mechanism of NLRP1 serving an instigator. The role of NLRP1 down-regulation in promoting tumorigenesis has also been accepted. Compared to healthy intestinal epithelial tissue, NLRP1 has been found to be down-regulated in colorectal cancer [21]. Moreover, the NLRP1 expression level reduced with the raising of colorectal cancer progression and the lowest level measured in stage III/IV patients [22]. In terms of lung tissue, the functional research of NLRP1 mainly focused on non-tumor diseases, such as: the NLRP1 inflammasome initiates pyroptosis, which subsequently leads to a self-amplifying cascade of cell injury within the lung [23]; the fine-tuning of the NLRP1 inflammasome plays an important role in maintaining the lung tissue integrity and treating the chronic inflammation of the airway [24]; the activation of the NLRP1 inflammasome pathways contributes to pulmonary fibrosis caused by latent MCMV infection in mice [25]. These studies did not involve the role of NLRP1 in lung tumors, but they confirmed that NLRP1 played a pivotal role in the lung inflammatory diseases. Moreover, NLRP3, another critical NLR family member, has been proved to modulate the progression of lung cancer, such as: tumor-derived exosomal TRIM59 activated NLRP3 inflammasome signaling pathway to promote lung cancer progression by IL-1β secretion [26]; NLRP3 could also promote metabolic reprogramming to regulate non-small cell lung cancer cell growth [27].

To acquire more detailed insights into the potential functions of NLRP1 in LUAD, we performed bioinformatic analysis of publicly available data. Analysis of transcriptome from more than 1,200 clinical samples derived from four geographic regions indicated that NLRP1 mRNA levels were obviously lower in LUAD tissue than in noncancerous lung tissue. Moreover, with the expression level of NLRP1 decreasing, the progression of LUAD got worsen, which specifically reflected on the advanced clinical characteristics of tumor pathological stages, lymph node metastasis status, and primary tumor status. These results indicate that NLRP1 played an inhibitory role in the LUAD progress. Here we noticed that the M stage representing the distant metastasis status, was not affected by the increasing of the NLRP1 expression level (Figure 2E). Combined with the subsequent analysis, we got known that NLRP1 mainly affected tumor progression by regulating the immune microenvironment in tumor tissues, which may not play an essential role in promoting tumor distant metastasis. Moreover, we also noticed there was a high amount of missing data regarding the patient’s M status, so the relationship between NLRP1 expression and M stage analyzed from this data was not accurate, and needs further confirmation.

As the body ages, humans suffer many diseases, including tumors. We tried to find the effect of the patients’ age on the expression level of NLRP1. First, we used the Spearman rank correlation coefficient to measure the degree of linear correlation between NLRP1 expression level and age. However, as shown in Supplementary Figure 1A, the degree of linear correlation between them was not high. Then we divided patients by the cut-off of age and used the Wilcoxon signed-rank test to evaluate the NLRP1 expression level of patients between age groups. To get the best cut-off of age, age groups were set by an interval for every 5 year ranging from 45 to 80 years (there were less than 10 patients under 40 years or over 85 years). In Supplementary Figure 1B, we clearly see that when patients’ NLRP1 expression level between age groups has a significant distinction, the maximum cut-off of age can be set at 55 years. Moreover, we found that the samples of elderly female patients had higher NLRP1 expression and as shown in Table 2, patients over 55 years old accounted for 83% in the TCGA-LUAD dataset, 55% of them are female patients, which is enough to demonstrate that LUAD is susceptible to the elderly. Although, as shown in Table 1, age and gender were not direct factors affecting the prognosis of LUAD patients in the TCGA-LUAD dataset. However, we believe that the differential expression of NLRP1 in age and gender groups has promising significance, which is worthy of further exploration. Moreover, multivariate analysis further showed that NLRP1 expression was an independent factor of LUAD patient prognosis. Therefore, our results indicated that the down-regulation of NLRP1 expression occurs in the majority of LUAD samples and participates in pathological progression. As a potential prognostic marker, NLRP1 deserves further clinical verification.

**Table 2:** TCGA lung adenocarcinoma patients characteristics.

**Clinical parameters**	**Variable**	**Total (458)**	**Percentages (%)**
Age	≤55	77	17%
	>55	381	83%
Gender	Female	250	55%
	Male	208	45%
T stage	T1	158	34%
	T2	239	52%
	T3	39	9%
	T4	19	4%
	Unknow	3	1%
N stage	N0	295	64%
	N1	151	33%
	Unknow	12	3%
M stage	M0	305	67%
	M1	24	5%
	Unknow	129	28%
TNM stage	Stage I	246	54%
	Stage II	105	23%
	Stage III	74	16%
	Stage IV	25	5%
	Unknow	8	2%
Survival status	Death	157	34%
	Alive	301	66%

The TCGAportal database provides additional information related to the differential expression of NLRP1. In the new subgroup system reclassified by histopathology, anatomy and mutation classification of LUAD, TRU subgroup with better prognostic results and PI subgroup with good immune activity had higher NLRP1 expression levels than PP subgroup [28]. This indicates that the expression of NLRP1 affected not only the prognosis but also the immune activation status of cancer tissues. There is a possibility that the expression of NLRP1 affected the prognosis of patients by affecting immune activity.

In addition, the mutation status of several cancer-related genes with high mutation probability in LUAD significantly affected the expression of NLRP1. In tissues with wild-type TP53, KRAS, KEAP1 or STK11, NLRP1 expression was relatively high. However, the expression level elevation of NLRP1 more appeared in tissues with EGFR mutation.

TP53 is the most frequently mutated gene in human cancer. The wild-type p53 protein acts as a stress sensor and regulates many cellular pathways, including cell cycle arrest, aging, apoptosis, metabolic changes, DNA repair, and other tumor suppression-related mechanisms [29]. Mutated p53 protein loses the cellular regulatory function of the wild-type p53 protein [30]. Beyond losing oncosuppressor activities, some mutant p53 proteins acquire oncogenic features that create overgrowth and survival advantages in cells [31]. Abnormal mutation of KRAS can initiate the activation of an array of signaling pathways and affect a series of essential cellular processes, such as cell differentiation, growth, chemotaxis and apoptosis [32]. Wild-type KRAS shows a tumor growth-inhibiting function in KRAS-mutant cancer by preventing cellular transformation and decreasing the tumor burden in several malignancies [33, 34]. The stress sensor KEAP1 and transcription factor NRF2 play a crucial cooperative role in cytoprotection against oxidative stress [35]. Thirty percent of human lung cancers develop mutations in either KEAP1 or NFE2I2, which promote metastasis in LUAD by leading to the stabilization of NRF2, the NFE2I2 gene product [36]. STK11 (serine/threonine kinase 11), also named LKB1 (liver kinase B1), is a tumor suppressor gene that regulates cellular metabolism/energy homeostasis, growth and polarity [37, 38]. Loss of normal activity can change into alterations of tumor-associated metabolism and differentiation [39–41] and positively impact on oncogenesis, local progression and metastatic dissemination [42]. EGFR plays crucial roles in the proliferation, growth, repair and survival of tumor cells [43]. EGFR is overexpressed in many tumors of epithelial origin, such as non-small cell lung cancer, breast cancer, glioma, head and neck cancer, cervical cancer, bladder cancer, and gastric cancer. In addition, abnormal expression of EGFR is closely related to neovascularization, tumor invasion and metastasis, tumor chemotherapy resistance and tumor prognosis [44, 45]. The effects of these mutant genes on NLRP1 give us reasons to believe that the differential expression of NLRP1 plays a regulatory role in the process of LUAD, which requires further study. Moreover, the expression level of NLRP1 was also affected by the DNA methylation level of multiple sites on chromosome 17.

NLRP1 has a significant impact on the prognosis of LUAD patients. Most genes co-expressed with NLRP1 in LUAD, whether positively or negatively related, had an obvious association with the prognosis of LUAD patients. Moreover, these co-expressed genes were considerably enriched in inflammation and immune-related pathways, which coincided with the known NLRP1 function. All signs indicated that NLRP1 may be involved in regulating the immune microenvironment to improve the prognosis of LUAD.

NLRP1 can assemble with the adaptor apoptosis-associated speck-like protein (ASC) and caspase-1 to regulate innate and adaptive immune responses by forming inflammasome [46]. In colon cancer, compared to wild type mice, the loss of NLRP1 can cause the increasing of tumorigenesis driven by inflammation, which has been proved in the Nlrp1b-/- mouse models [47]. In this model, epithelial cell-derived IL-18 reduction and immune system homeostasis weakening in the colon could be the explanation for promoting tumor progression.

The analysis results generated with the TIMER database showed this inference more visually: the expression levels of NLRP1 in the TCGA-LUAD and GSE72094 datasets had significant positive correlations with the levels of infiltrated B cells, CD4 T cells, CD8 T cells, dendritic cells, macrophages and neutrophils. In addition, the expression of NLRP1 exhibited a significant positive correlation with the immune score of LUAD samples based on the ESTIMATE algorithm. Moreover, patients with higher immune score or NLRP1 expression had longer survival time than patients with lower one. These results clearly show that NLRP1 expression can improve the immune microenvironment of tumors in LUAD patients by increasing the infiltration of immune cells, thereby improving patient prognosis. TIMER database analysis also showed that the expression level of NLRP1 could affect the immune cell infiltration level in multiple cancer tissues, including adrenocortical carcinoma, breast invasive carcinoma, bladder urothelial carcinoma, cholangiocarcinoma, colon adenocarcinoma, kidney chromophore, kidney renal papillary cell carcinoma, brain lower grade glioma, pancreatic adenocarcinoma, pheochromocytoma and paraganglioma, rectum adenocarcinoma, stomach adenocarcinoma, testicular germ cell tumors, thyroid carcinoma, skin cutaneous melanoma, uterine carcinosarcoma and uterine corpus endometrial carcinoma (Supplementary Figure 3). Moreover, we assessed the correlation between NLRP1 and the immune system via the TISIDB database, and the results showed that NLRP1 regulated various immune molecules.

Inevitably, this research has one inherent limitation that needs to be addressed. Because this research was based on data onto the TCGA and GEO datasets and has not been validated in the prospective clinical trial, the mechanism of NLRP1 in LUAD is still unclear. Although there was this drawback, the results of our study still suggested that the decreasing NLRP1 expression could be applied as a reliable predictor of LUAD progress.

We concluded that there is a possible prognostic molecular marker for poor survival in LUAD, called NLRP1 expression. Decreased NLRP1 expression led to worsening of clinical features (pathological stage of tumor, lymph node metastasis status, primary tumor status and prognosis). NLR family has been verified to be involved in the regulation of various immune cells’ migration. In this study, we found that the expression level of NLRP1 was significantly associated with the infiltration level of various TIICs in LUAD tissues. The immune microenvironment, composed of these TIICs, profoundly affected the prognosis of LUAD. Therefore, in our clinical work, after measuring the NLRP1 expression level in the surgical specimens of LUAD patients, we can use it to evaluate the malignancy of tumor, predict the prognosis of patient, assess the status of immune microenvironment in tumor tissue, and even develop immunotherapeutic drugs targeting NLRP1, which has been verified to be a right direction: DAC (5-aza-2-deoxycytidine), an antitumor drug, can restore the NLRP1 expression level to suppress the growth of colon cancer [21]. We recommend strongly that researchers in the field of tumor immunology conduct further research on NLRP1 in LUAD to gradually elaborate the biological role of NLRP1 in the immune microenvironment and prognosis of LUAD patients.

## MATERIALS AND METHODS

### Data acquisition and processing

Gene expression profiles of LUAD patients and clinical patient data were downloaded from the TCGA (https://portal.gdc.cancer.gov/) database [48] on December 16, 2019. Tumor samples without clinical data were removed. 458 LUAD samples and 54 noncancerous adjacent tissue samples were included in this research. Furthermore, to exclude the impact of other factors on the survival probability of patients, tumor samples with the follow-up period of less than 30 days were removed in prognostic analysis.

Seven sets of LUAD chip datasets GSE116959, GSE32863, GSE43458, GSE7670, GSE83213, GSE75073 and GSE72094 were downloaded from the GEO (https://www.ncbi.nlm.nih.gov/geo/) database [49]. Of the seven datasets, GSE72094 contains detailed clinical prognostic information, so it was utilized as a validation set in the study, and the other six sets of data were used to study the differential expression of genes. For the probe data, the probe was mapped to the GeneSymbol using the platform of each chip dataset based on. GSE83213 and GSE72094 were based on the GPL10558 platform, GSE116959 was based on the GPL17077 platform, GSE43458 was based on the GPL6244 platform, GSE75037 and GSE32863 were based on the GPL68848 platform and GSE10072 and GSE7670 were based on the GPL96 platform. The detailed information of GEO datasets was presented in Table 3.

**Table 3:** GEO lung adenocarcinoma patient datasets characteristics.

**Dataset-ID**	**LUAD**	**Normal**	**Region**	**Organization/Lab**	**Platforms**
GSE10072	58	49	USA	National Cancer Institute, NIH	GPL96
GSE116959	57	11	France	Functional Genomics Platform of Nice-Sophia-Antipolis	GPL17077
GSE43458	80	30	USA	University of Texas MD Anderson Cancer Center	GPL6244
GSE7670	30	29	Taiwan	Taipei Veterans General Hospital	GPL96
GSE83213	11	46	Canada	Laval University	GPL10558
GSE32863	58	58	USA	University of Southern California	GPL6884
GSE75037	83	83	USA	UT Southwestern Medical Center	GPL6884
GSE72094	442	0	USA	Moffitt Cancer Center	GPL15048

### Differential expression analysis of NLRP1

GSE10072, GSE116959, GSE43458, GSE7670, GSE83213, GSE75037, GSE32863 and TCGA were used to evaluate the different expression level of NLRP1 in LUAD and normal tissue. The relationship between NLRP1 expression and clinical parameters including age, gender, T stage, N stage, and M stage and TNM stage were then evaluated in TCGA LUAD dataset. The TCGAportal (http://tumorsurvival.org/) database was used to determine the association between NLRP1 expression and tumor clinical subtypes, methylation, and common mutant genes.

### LinkedOmics database analysis

The LinkedOmics (http://www.linkedomics.org/login.php) database is a web platform for studying multi-dimensional data sets related to 32 cancers in TCGA [50]. Firstly, all genes co-expression with NLRP1 were obtained and presented by volcano figure. Then heatmaps were used to respectively show the 50 highest correlated genes and their prognostic significance in LUAD. Finally, function module analysis of GO_BP (Gene Ontology Biological Process), KEGG (Kyoto Encyclopedia of Genes and Genomes) pathways by GSEA (Gene Set Enrichment Analysis). According to the rank criterion was FDR (false discovery rate) < 0.05, simulations were performed for 1000 times.

### TIMER and ESTIMATE database analysis

TIMER (The Tumor IMmune Estimation Resource) (https://cistrome.shinyapps.io/timer/) database is a comprehensive resource for systematic analysis of immune infiltrates across multiple cancer types [51], which includes 32 cancer types. We used the deconvolution method provided by TIMER to infer the abundance of TIICs (tumor-infiltrating immune cells) from the gene expression profiles of the LUAD samples in the TCGA and GEO dataset.

ESTIMATE (The Estimation of STromal and Immune cells in MAlignant Tumor tissues using Expression data) (https://bioinformatics.mdanderson.org/public-software/estimate/) database is a tool for predicting tumor purity and the presence of infiltrating stromal/immune cells in tumor tissue using gene expression data [52]. The ESTIMATE algorithm is based on single-sample GSEA and generates immune score, which represents the degrees of infiltration of immune cells in tumor tissue.

### TISIDB database analysis

The TISIDB (http://cis.hku.hk/TISIDB) database integrates 988 reported immune-related antitumor genes [53], high-throughput screening techniques, molecular profiling, and para-cancerous multiomic data, as well as various resources for immunological data obtained from seven public databases. TISIDB enables analysis of associations between NLRP1 and lymphocytes, immunomodulators, and chemokines.

### Statistical analysis

All statistical analyses were performed through R 3.5.2 Software. A p-value < 0.05 was considered statistically significant. The relationship between NLRP1 expression level and clinical parameters was analyzed via the Wilcoxon signed-rank test, Wilcoxon rank sum test, Kruskal-Wallis rank sum test. Univariate and multivariate Cox regressions were implemented to investigate the prognostic impact of NLRP1 in LUAD patients of TCGA dataset. The Kaplan-Meier method was used to verify the relationship between expression levels of NLRP1, and patients’ OS in TCGA and GEO LUAD datasets. Spearman rank correlation tests were used to assess the correlation between NLRP1 and immune signatures gotten from the TIMER, ESTIMATH and TISIDB databases.

## Supplementary Material

Supplementary Figures

Supplementary Table 1
