# Supplementary material for: Low expression of NLRP1 is associated with a poor prognosis and immune infiltration in lung adenocarcinoma patients
Source: Aging (Albany NY). 2021 Mar 3;13(5):7570–88. doi: 10.18632/aging.202620 (PMC7993699; doi:10.18632/aging.202620)
Supplement: Supplementary Figures [file aging-13-202620-s001.pdf]

SUPPLEMENTARY FIGURES

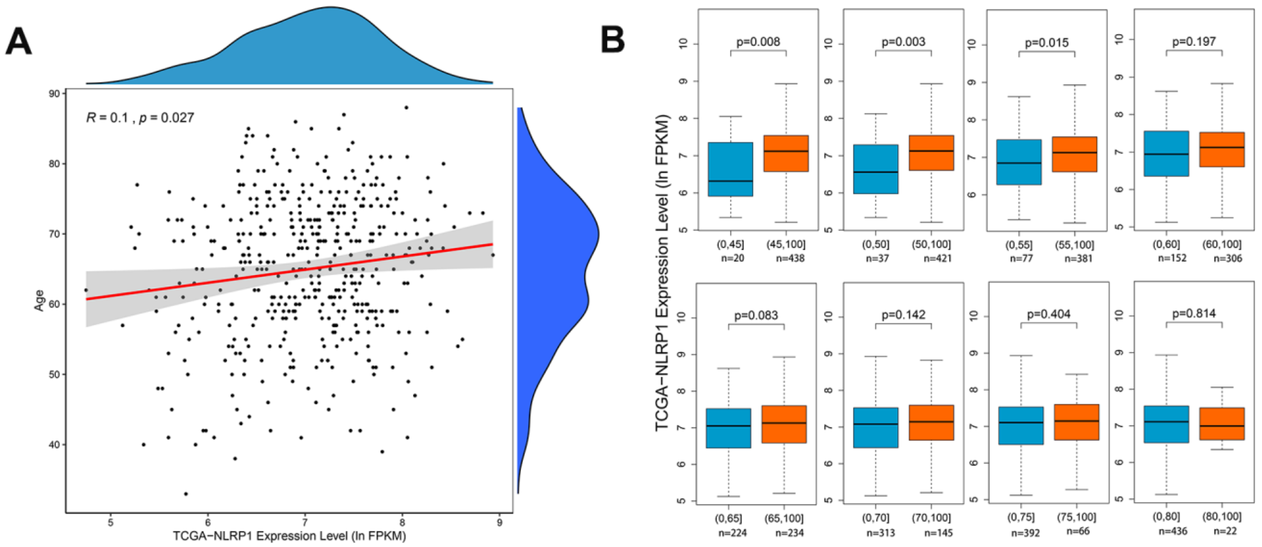

Supplementary Figure 1. (**A**) Correlation of NLRP1 expression with patients' age in LUAD. (**B**) Relations between different age groups and NLRP1 expression.

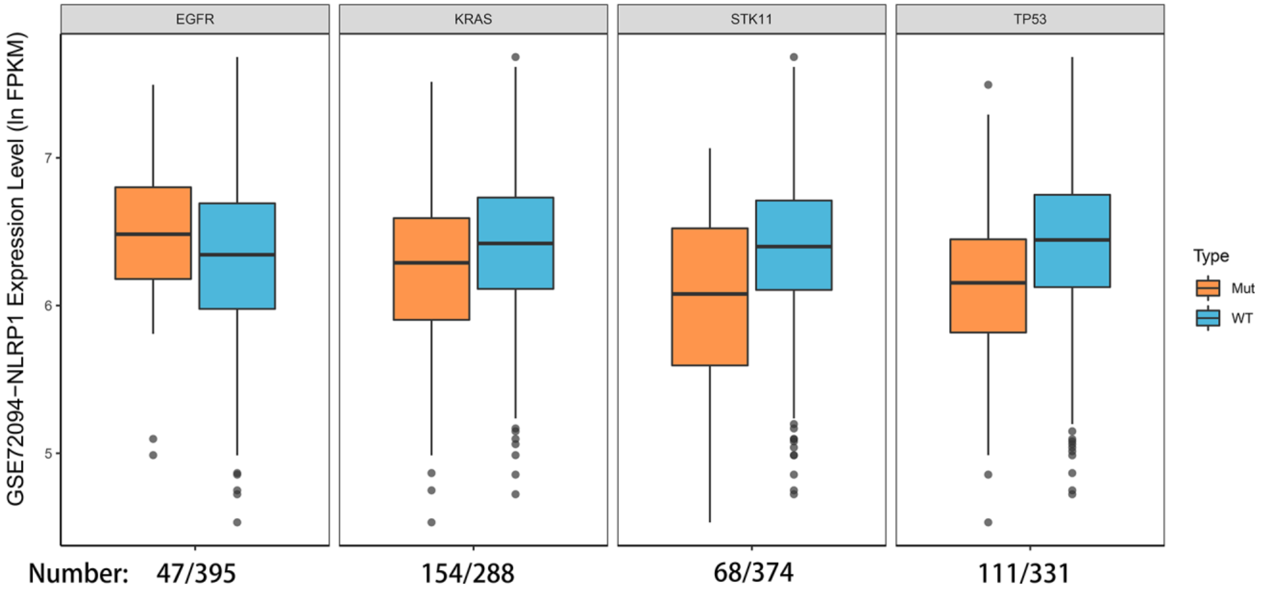

Supplementary Figure 2. In the verification dataset GSE72094, the expression level of NLRP1 was influenced by the mutation status of TP53, KRAS, STK11 and EGFR.

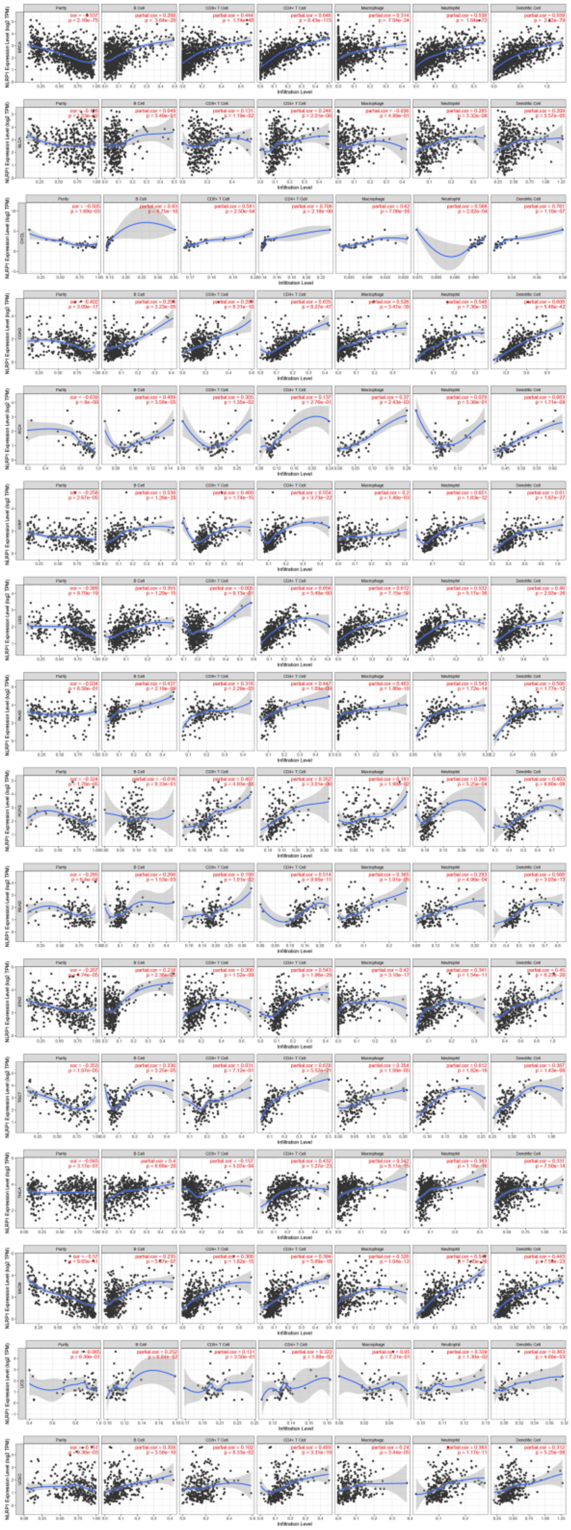

Supplementary Figure 3. TIMER database analysis showed that the expression level of NLRP1 could affect the immune cell infiltration levels in multiple cancers, including ACC (adrenocortical carcinoma), BRCA (breast invasive carcinoma), BLCA (bladder urothelial carcinoma), CHOL (cholangiocarcinoma), COAD (colon adenocarcinoma), KICH (kidney chromophore), KIRP (kidney renal papillary cell carcinoma), LGG (brain lower grade glioma), PAAD (pancreatic adenocarcinoma), PCPG (pheochromocytoma and paraganglioma), READ (rectum adenocarcinoma), STAD (stomach adenocarcinoma), TGCT (testicular germ cell tumors), THCA (thyroid carcinoma), SKCM (skin cutaneous melanoma), UCS (uterine carcinosarcoma) and UCEC (uterine corpus endometrial carcinoma).
