# Supplementary material for: Low expression of NLRP1 is associated with a poor prognosis and immune infiltration in lung adenocarcinoma patients
Source: Aging (Albany NY). 2021 Mar 3;13(5):7570–88. doi: 10.18632/aging.202620 (PMC7993699; doi:10.18632/aging.202620)
Supplement: Supplementary Table 1 [file aging-13-202620-s002.pdf]

SUPPLEMENTARY TABLE

Supplementary Table 1. Multivariate cox regression analyses for OS in LUAD patients (with *p*-adjust).

Variables	Multivariate analysis			
	**No. of patients**	**HR (95% CI)**	***p*-value**	***p*-adjust (*FDR*)**
T stage				
T1/2 vs.3/4	265/41	1.255(0.967−1.629)	0.087	0.145
N stage				
N0 vs. N1	195/111	2.123(1.348−3.345)	0.001	0.005
M stage				
M0 vs. M1	286/20	1.121(0.570−2.206)	0.741	0.741
Pathological stage				
Stage I/II vs. III/IV	231/75	1.521(0.880−2.629)	0.133	0.166
NLRP1				
High vs. low expression	153/153	0.713(0.542−0.939)	0.016	0.040

FDR, false discovery rate; OS, Overall Survival.
